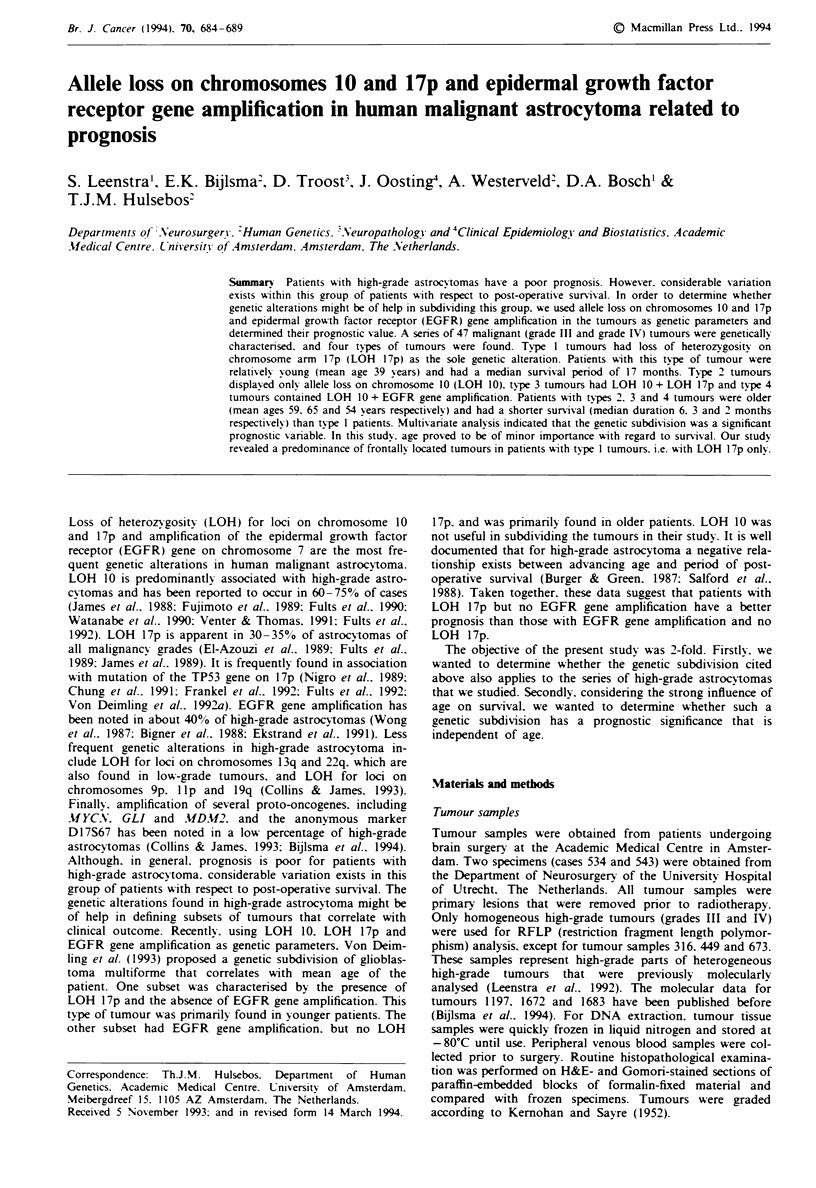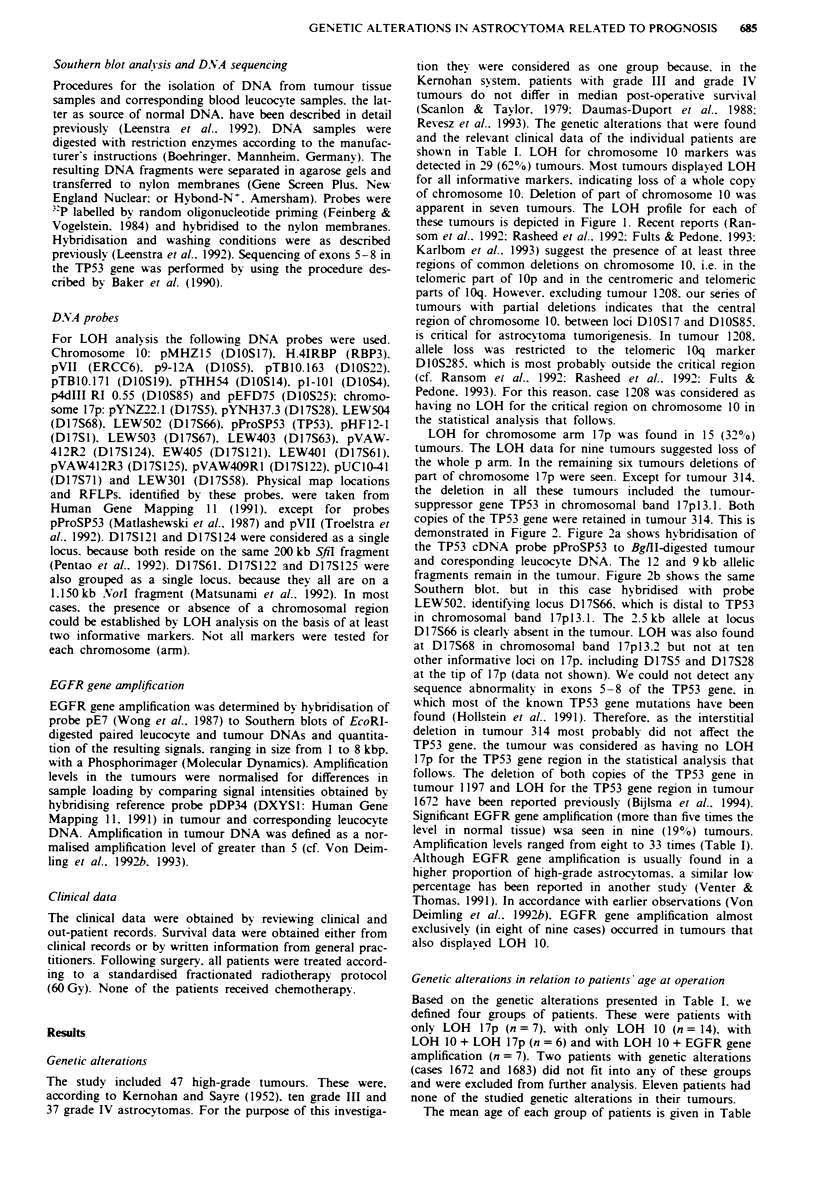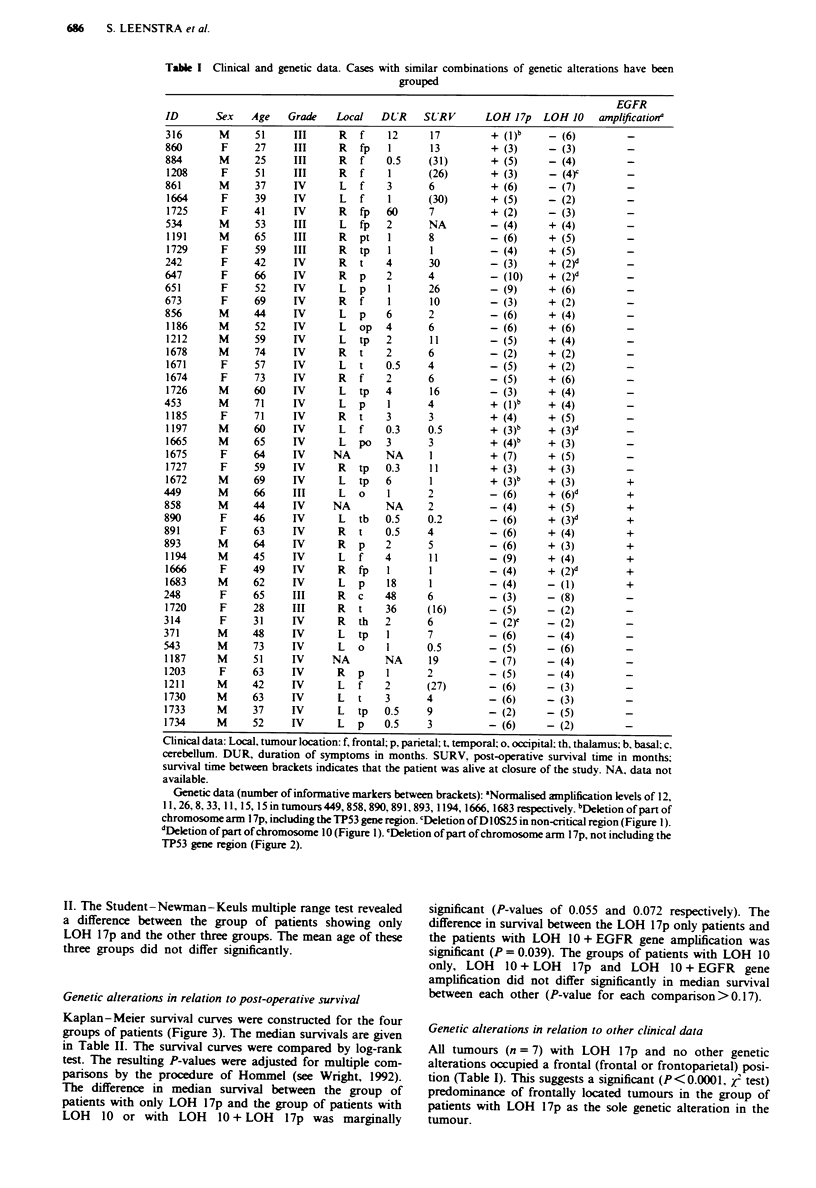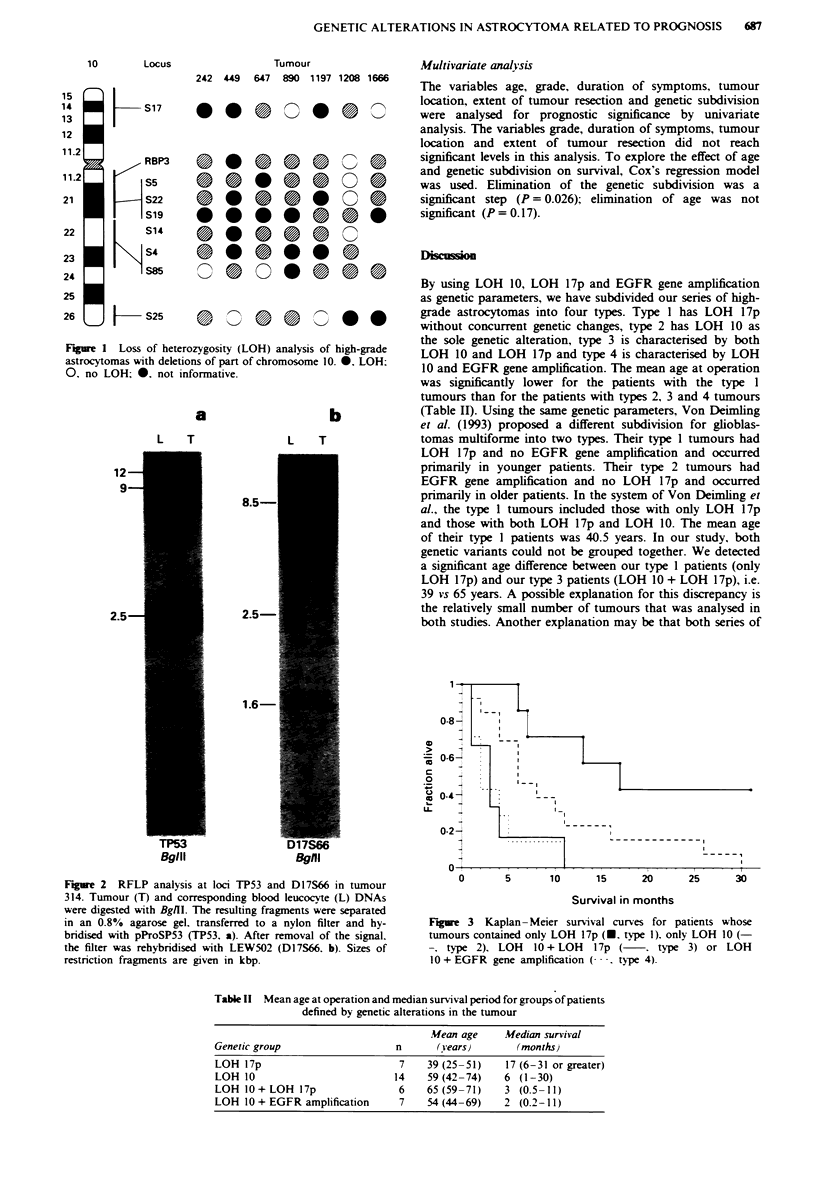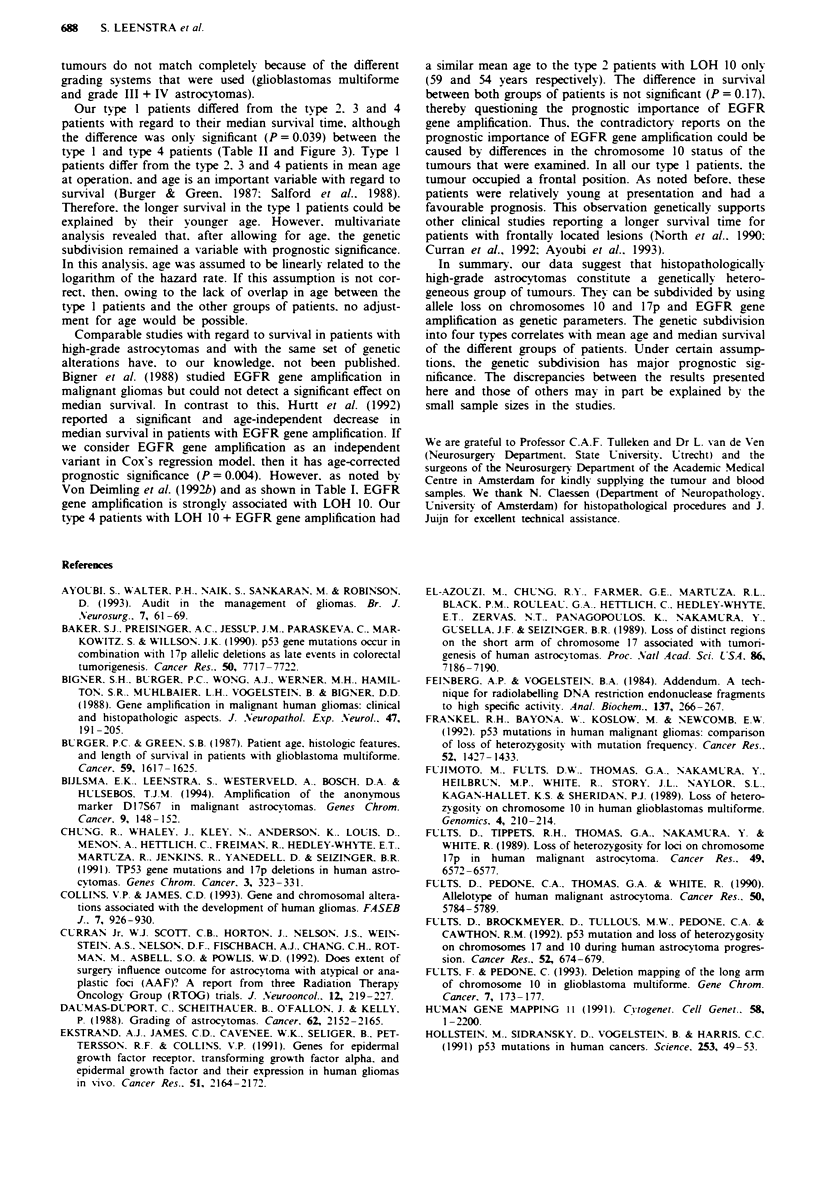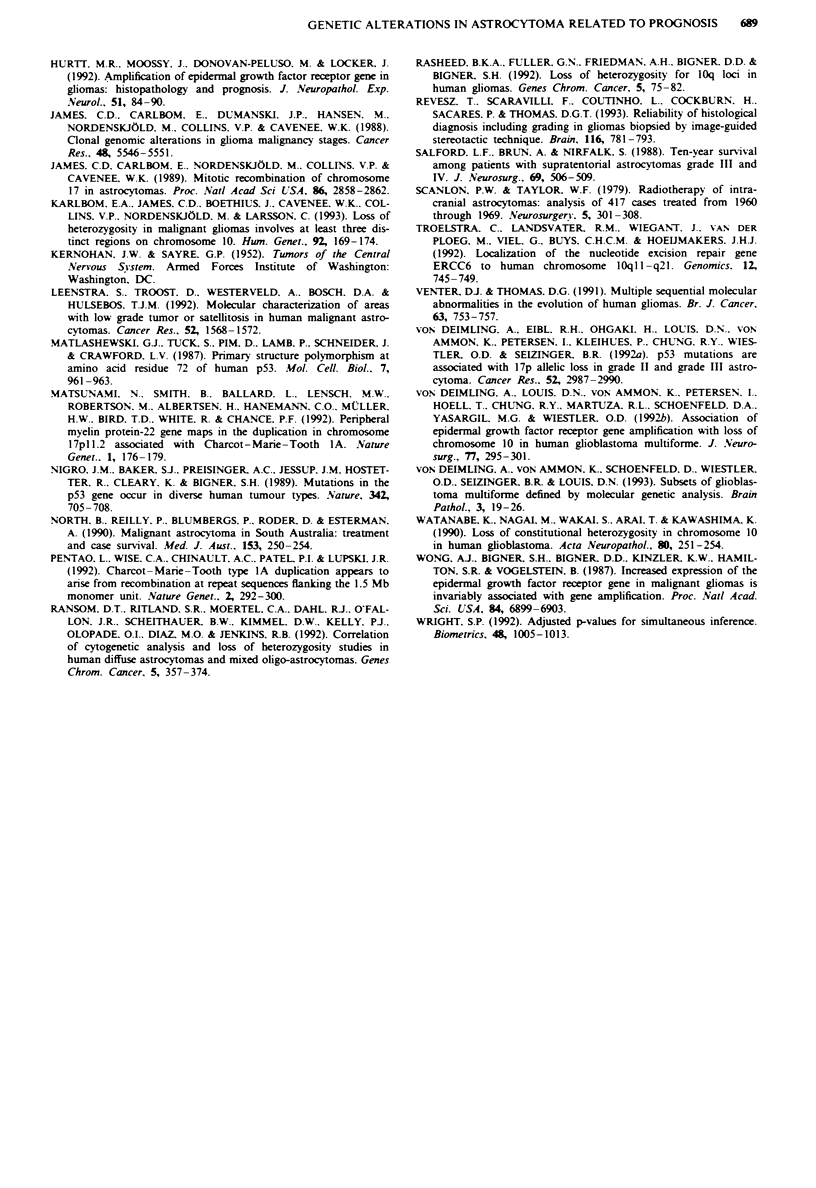# Allele loss on chromosomes 10 and 17p and epidermal growth factor receptor gene amplification in human malignant astrocytoma related to prognosis.

**DOI:** 10.1038/bjc.1994.373

**Published:** 1994-10

**Authors:** S. Leenstra, E. K. Bijlsma, D. Troost, J. Oosting, A. Westerveld, D. A. Bosch, T. J. Hulsebos

**Affiliations:** Department of Neurosurgery, University of Amsterdam, The Netherlands.

## Abstract

**Images:**


Br. J. Cancer (1994). 70, 684-689 © Macmillan Press Ltd., 1994

### Allele loss on chromosomes 10 and 17p and epidermal growth factor receptor gene amplification in human malignant astrocytoma related to prognosis

S. Leenstra[1], E.K. Bijlsma[2], D. Troost[3], J. Oosting[4], A. Westerveld[2], D.A. Bosch[1] & T.J.M. Hulsebos[2]

*Departments of [1]Neurosurgery, [2]Human Genetics, [3]Neuropathology and [4]Clinical Epidemiology and Biostatistics, Academic Medical Centre, University of Amsterdam, Amsterdam, The Netherlands.*

**Summary** Patients with high-grade astrocytomas have a poor prognosis. However, considerable variation exists within this group of patients with respect to post-operative survival. In order to determine whether genetic alterations might be of help in subdividing this group, we used allele loss on chromosomes 10 and 17p and epidermal growth factor receptor (EGFR) gene amplification in the tumours as genetic parameters and determined their prognostic value. A series of 47 malignant (grade III and grade IV) tumours were genetically characterised, and four types of tumours were found. Type 1 tumours had loss of heterozygosity on chromosome arm 17p (LOH 17p) as the sole genetic alteration. Patients with this type of tumour were relatively young (mean age 39 years) and had a median survival period of 17 months. Type 2 tumours displayed only allele loss on chromosome 10 (LOH 10), type 3 tumours had LOH 10 + LOH 17p and type 4 tumours contained LOH 10 + EGFR gene amplification. Patients with types 2, 3 and 4 tumours were older (mean ages 59, 65 and 54 years respectively) and had a shorter survival (median duration 6, 3 and 2 months respectively) than type 1 patients. Multivariate analysis indicated that the genetic subdivision was a significant prognostic variable. In this study, age proved to be of minor importance with regard to survival. Our study revealed a predominance of frontally located tumours in patients with type 1 tumours, i.e. with LOH 17p only.

Loss of heterozygosity (LOH) for loci on chromosome 10 and 17p and amplification of the epidermal growth factor receptor (EGFR) gene on chromosome 7 are the most frequent genetic alterations in human malignant astrocytoma. LOH 10 is predominantly associated with high-grade astrocytomas and has been reported to occur in 60–75% of cases (James *et al.*, 1988; Fujimoto *et al.*, 1989; Fults *et al.*, 1990; Watanabe *et al.*, 1990; Venter & Thomas, 1991; Fults *et al.*, 1992). LOH 17p is apparent in 30–35% of astrocytomas of all malignancy grades (El-Azouzi *et al.*, 1989; Fults *et al.*, 1989; James *et al.*, 1989). It is frequently found in association with mutation of the TP53 gene on 17p (Nigro *et al.*, 1989; Chung *et al.*, 1991; Frankel *et al.*, 1992; Fults *et al.*, 1992; Von Deimling *et al.*, 1992*a*). EGFR gene amplification has been noted in about 40% of high-grade astrocytomas (Wong *et al.*, 1987; Bigner *et al.*, 1988; Ekstrand *et al.*, 1991). Less frequent genetic alterations in high-grade astrocytoma include LOH for loci on chromosomes 13q and 22q, which are also found in low-grade tumours, and LOH for loci on chromosomes 9p, 11p and 19q (Collins & James, 1993). Finally, amplification of several proto-oncogenes, including *MYCN*, *GLI* and *MDM2*, and the anonymous marker D17S67 has been noted in a low percentage of high-grade astrocytomas (Collins & James, 1993; Bijlsma *et al.*, 1994). Although, in general, prognosis is poor for patients with high-grade astrocytoma, considerable variation exists in this group of patients with respect to post-operative survival. The genetic alterations found in high-grade astrocytoma might be of help in defining subsets of tumours that correlate with clinical outcome. Recently, using LOH 10, LOH 17p and EGFR gene amplification as genetic parameters, Von Deimling *et al.* (1993) proposed a genetic subdivision of glioblastoma multiforme that correlates with mean age of the patient. One subset was characterised by the presence of LOH 17p and the absence of EGFR gene amplification. This type of tumour was primarily found in younger patients. The other subset had EGFR gene amplification, but no LOH 17p, and was primarily found in older patients. LOH 10 was not useful in subdividing the tumours in their study. It is well documented that for high-grade astrocytoma a negative relationship exists between advancing age and period of post-operative survival (Burger & Green, 1987; Salford *et al.*, 1988). Taken together, these data suggest that patients with LOH 17p but no EGFR gene amplification have a better prognosis than those with EGFR gene amplification and no LOH 17p.

The objective of the present study was 2-fold. Firstly, we wanted to determine whether the genetic subdivision cited above also applies to the series of high-grade astrocytomas that we studied. Secondly, considering the strong influence of age on survival, we wanted to determine whether such a genetic subdivision has a prognostic significance that is independent of age.

Correspondence: Th.J.M. Hulsebos, Department of Human Genetics, Academic Medical Centre, University of Amsterdam, Meibergdreef 15, 1105 AZ Amsterdam, The Netherlands.
Received 5 November 1993; and in revised form 14 March 1994.

### Materials and methods

### Tumour samples

Tumour samples were obtained from patients undergoing brain surgery at the Academic Medical Centre in Amsterdam. Two specimens (cases 534 and 543) were obtained from the Department of Neurosurgery of the University Hospital of Utrecht, The Netherlands. All tumour samples were primary lesions that were removed prior to radiotherapy. Only homogeneous high-grade tumours (grades III and IV) were used for RFLP (restriction fragment length polymorphism) analysis, except for tumour samples 316, 449 and 673. These samples represent high-grade parts of heterogeneous high-grade tumours that were previously molecularly analysed (Leenstra *et al.*, 1992). The molecular data for tumours 1197, 1672 and 1683 have been published before (Bijlsma *et al.*, 1994). For DNA extraction, tumour tissue samples were quickly frozen in liquid nitrogen and stored at −80°C until use. Peripheral venous blood samples were collected prior to surgery. Routine histopathological examination was performed on H&E- and Gomori-stained sections of paraffin-embedded blocks of formalin-fixed material and compared with frozen specimens. Tumours were graded according to Kernohan and Sayre (1952).

*Southern blot analysis and DNA sequencing*

Procedures for the isolation of DNA from tumour tissue samples and corresponding blood leucocyte samples, the latter as source of normal DNA, have been described in detail previously (Leenstra *et al.*, 1992). DNA samples were digested with restriction enzymes according to the manufacturer's instructions (Boehringer, Mannheim, Germany). The resulting DNA fragments were separated in agarose gels and transferred to nylon membranes (Gene Screen Plus, New England Nuclear; or Hybond-N$^+$, Amersham). Probes were $^{32}$P labelled by random oligonucleotide priming (Feinberg & Vogelstein, 1984) and hybridised to the nylon membranes. Hybridisation and washing conditions were as described previously (Leenstra *et al.*, 1992). Sequencing of exons 5–8 in the TP53 gene was performed by using the procedure described by Baker *et al.* (1990).

*DNA probes*

For LOH analysis the following DNA probes were used. Chromosome 10: pMHZ15 (D10S17), H.4IRBP (RBP3), pVII (ERCC6), p9-12A (D10S5), pTB10.163 (D10S22), pTB10.171 (D10S19), pTHH54 (D10S14), p1-101 (D10S4), p4dIII RI 0.55 (D10S85) and pEFD75 (D10S25); chromosome 17p: pYNZ22.1 (D17S5), pYNH37.3 (D17S28), LEW504 (D17S68), LEW502 (D17S66), pProSP53 (TP53), pHF12-1 (D17S1), LEW503 (D17S67), LEW403 (D17S63), pVAW-412R2 (D17S124), EW405 (D17S121), LEW401 (D17S61), pVAW412R3 (D17S125), pVAW409R1 (D17S122), pUC10-41 (D17S71) and LEW301 (D17S58). Physical map locations and RFLPs, identified by these probes, were taken from Human Gene Mapping 11 (1991), except for probes pProSP53 (Matlashewski *et al.*, 1987) and pVII (Troelstra *et al.*, 1992). D17S121 and D17S124 were considered as a single locus, because both reside on the same 200 kb *Sfi*I fragment (Pentao *et al.*, 1992). D17S61, D17S122 and D17S125 were also grouped as a single locus, because they all are on a 1,150 kb *Not*I fragment (Matsunami *et al.*, 1992). In most cases, the presence or absence of a chromosomal region could be established by LOH analysis on the basis of at least two informative markers. Not all markers were tested for each chromosome (arm).

*EGFR gene amplification*

EGFR gene amplification was determined by hybridisation of probe pE7 (Wong *et al.*, 1987) to Southern blots of *Eco*RI-digested paired leucocyte and tumour DNAs and quantitation of the resulting signals, ranging in size from 1 to 8 kbp, with a Phosphorimager (Molecular Dynamics). Amplification levels in the tumours were normalised for differences in sample loading by comparing signal intensities obtained by hybridising reference probe pDP34 (DXYS1: Human Gene Mapping 11, 1991) in tumour and corresponding leucocyte DNA. Amplification in tumour DNA was defined as a normalised amplification level of greater than 5 (cf. Von Deimling *et al.*, 1992*b*, 1993).

*Clinical data*

The clinical data were obtained by reviewing clinical and out-patient records. Survival data were obtained either from clinical records or by written information from general practitioners. Following surgery, all patients were treated according to a standardised fractionated radiotherapy protocol (60 Gy). None of the patients received chemotherapy.

**Results**

*Genetic alterations*

The study included 47 high-grade tumours. These were, according to Kernohan and Sayre (1952), ten grade III and 37 grade IV astrocytomas. For the purpose of this investigation they were considered as one group because, in the Kernohan system, patients with grade III and grade IV tumours do not differ in median post-operative survival (Scanlon & Taylor, 1979; Daumas-Duport *et al.*, 1988; Revesz *et al.*, 1993). The genetic alterations that were found and the relevant clinical data of the individual patients are shown in Table I. LOH for chromosome 10 markers was detected in 29 (62%) tumours. Most tumours displayed LOH for all informative markers, indicating loss of a whole copy of chromosome 10. Deletion of part of chromosome 10 was apparent in seven tumours. The LOH profile for each of these tumours is depicted in Figure 1. Recent reports (Ransom *et al.*, 1992; Rasheed *et al.*, 1992; Fults & Pedone, 1993; Karlbom *et al.*, 1993) suggest the presence of at least three regions of common deletions on chromosome 10, i.e. in the telomeric part of 10p and in the centromeric and telomeric parts of 10q. However, excluding tumour 1208, our series of tumours with partial deletions indicates that the central region of chromosome 10, between loci D10S17 and D10S85, is critical for astrocytoma tumorigenesis. In tumour 1208, allele loss was restricted to the telomeric 10q marker D10S285, which is most probably outside the critical region (cf. Ransom *et al.*, 1992; Rasheed *et al.*, 1992; Fults & Pedone, 1993). For this reason, case 1208 was considered as having no LOH for the critical region on chromosome 10 in the statistical analysis that follows.

LOH for chromosome arm 17p was found in 15 (32%) tumours. The LOH data for nine tumours suggested loss of the whole p arm. In the remaining six tumours deletions of part of chromosome 17p were seen. Except for tumour 314, the deletion in all these tumours included the tumour-suppressor gene TP53 in chromosomal band 17p13.1. Both copies of the TP53 gene were retained in tumour 314. This is demonstrated in Figure 2. Figure 2a shows hybridisation of the TP53 cDNA probe pProSP53 to *Bgl*II-digested tumour and coresponding leucocyte DNA. The 12 and 9 kb allelic fragments remain in the tumour. Figure 2b shows the same Southern blot, but in this case hybridised with probe LEW502, identifying locus D17S66, which is distal to TP53 in chromosomal band 17p13.1. The 2.5 kb allele at locus D17S66 is clearly absent in the tumour. LOH was also found at D17S68 in chromosomal band 17p13.2 but not at ten other informative loci on 17p, including D17S5 and D17S28 at the tip of 17p (data not shown). We could not detect any sequence abnormality in exons 5–8 of the TP53 gene, in which most of the known TP53 gene mutations have been found (Hollstein *et al.*, 1991). Therefore, as the interstitial deletion in tumour 314 most probably did not affect the TP53 gene, the tumour was considered as having no LOH 17p for the TP53 gene region in the statistical analysis that follows. The deletion of both copies of the TP53 gene in tumour 1197 and LOH for the TP53 gene region in tumour 1672 have been reported previously (Bijlsma *et al.*, 1994). Significant EGFR gene amplification (more than five times the level in normal tissue) wsa seen in nine (19%) tumours. Amplification levels ranged from eight to 33 times (Table I). Although EGFR gene amplification is usually found in a higher proportion of high-grade astrocytomas, a similar low percentage has been reported in another study (Venter & Thomas, 1991). In accordance with earlier observations (Von Deimling *et al.*, 1992*b*), EGFR gene amplification almost exclusively (in eight of nine cases) occurred in tumours that also displayed LOH 10.

*Genetic alterations in relation to patients' age at operation*

Based on the genetic alterations presented in Table I, we defined four groups of patients. These were patients with only LOH 17p ($n = 7$), with only LOH 10 ($n = 14$), with LOH 10 + LOH 17p ($n = 6$) and with LOH 10 + EGFR gene amplification ($n = 7$). Two patients with genetic alterations (cases 1672 and 1683) did not fit into any of these groups and were excluded from further analysis. Eleven patients had none of the studied genetic alterations in their tumours.

The mean age of each group of patients is given in Table

**Table I** Clinical and genetic data. Cases with similar combinations of genetic alterations have been grouped

| *ID* | *Sex* | *Age* | *Grade* | *Local* | *DUR* | *SURV* | *LOH 17p* | *LOH 10* | *EGFR amplification*[a] |
|---|---|---|---|---|---|---|---|---|---|
| 316 | M | 51 | III | R f | 12 | 17 | + (1)[b] | − (6) | − |
| 860 | F | 27 | III | R fp | 1 | 13 | + (3) | − (3) | − |
| 884 | M | 25 | III | R f | 0.5 | (31) | + (5) | − (4) | − |
| 1208 | F | 51 | III | R f | 1 | (26) | + (3) | − (4)[c] | − |
| 861 | M | 37 | IV | L f | 3 | 6 | + (6) | − (7) | − |
| 1664 | F | 39 | IV | L f | 1 | (30) | + (5) | − (2) | − |
| 1725 | F | 41 | IV | R fp | 60 | 7 | + (2) | − (3) | − |
| 534 | M | 53 | III | L fp | 2 | NA | − (4) | + (4) | − |
| 1191 | M | 65 | III | R pt | 1 | 8 | − (6) | + (5) | − |
| 1729 | F | 59 | III | R tp | 1 | 1 | − (4) | + (5) | − |
| 242 | F | 42 | IV | R t | 4 | 30 | − (3) | + (2)[d] | − |
| 647 | F | 66 | IV | R p | 2 | 4 | − (10) | + (2)[d] | − |
| 651 | F | 52 | IV | L p | 1 | 26 | − (9) | + (6) | − |
| 673 | F | 69 | IV | R f | 1 | 10 | − (3) | + (2) | − |
| 856 | M | 44 | IV | L p | 6 | 2 | − (6) | + (4) | − |
| 1186 | M | 52 | IV | L op | 4 | 6 | − (6) | + (6) | − |
| 1212 | M | 59 | IV | L tp | 2 | 11 | − (5) | + (4) | − |
| 1678 | M | 74 | IV | R t | 2 | 6 | − (2) | + (2) | − |
| 1671 | F | 57 | IV | L t | 0.5 | 4 | − (5) | + (2) | − |
| 1674 | F | 73 | IV | R f | 2 | 6 | − (5) | + (6) | − |
| 1726 | M | 60 | IV | L tp | 4 | 16 | − (3) | + (4) | − |
| 453 | M | 71 | IV | L p | 1 | 4 | + (1)[b] | + (4) | − |
| 1185 | F | 71 | IV | R t | 3 | 3 | + (4) | + (5) | − |
| 1197 | M | 60 | IV | L f | 0.3 | 0.5 | + (3)[b] | + (3)[d] | − |
| 1665 | M | 65 | IV | L po | 3 | 3 | + (4)[b] | + (3) | − |
| 1675 | F | 64 | IV | NA | NA | 1 | + (7) | + (5) | − |
| 1727 | F | 59 | IV | R tp | 0.3 | 11 | + (3) | + (3) | − |
| 1672 | M | 69 | IV | L tp | 6 | 1 | + (3)[b] | + (3) | + |
| 449 | M | 66 | III | L o | 1 | 2 | − (6) | + (6)[d] | + |
| 858 | M | 44 | IV | NA | NA | 2 | − (4) | + (5) | + |
| 890 | F | 46 | IV | L tb | 0.5 | 0.2 | − (6) | + (3)[d] | + |
| 891 | F | 63 | IV | R t | 0.5 | 4 | − (6) | + (4) | + |
| 893 | M | 64 | IV | R p | 2 | 5 | − (6) | + (3) | + |
| 1194 | M | 45 | IV | L f | 4 | 11 | − (9) | + (4) | + |
| 1666 | F | 49 | IV | R fp | 1 | 1 | − (4) | + (2)[d] | + |
| 1683 | M | 62 | IV | L p | 18 | 1 | − (4) | − (1) | + |
| 248 | F | 65 | III | R c | 48 | 6 | − (3) | − (8) | − |
| 1720 | F | 28 | III | R t | 36 | (16) | − (5) | − (2) | − |
| 314 | F | 31 | IV | R th | 2 | 6 | − (2)[e] | − (2) | − |
| 371 | M | 48 | IV | L tp | 1 | 7 | − (6) | − (4) | − |
| 543 | M | 73 | IV | L o | 1 | 0.5 | − (5) | − (6) | − |
| 1187 | M | 51 | IV | NA | NA | 19 | − (7) | − (4) | − |
| 1203 | F | 63 | IV | R p | 1 | 2 | − (5) | − (4) | − |
| 1211 | M | 42 | IV | L f | 2 | (27) | − (6) | − (3) | − |
| 1730 | M | 63 | IV | L t | 3 | 4 | − (6) | − (3) | − |
| 1733 | M | 37 | IV | L tp | 0.5 | 9 | − (2) | − (5) | − |
| 1734 | M | 52 | IV | L p | 0.5 | 3 | − (6) | − (2) | − |

Clinical data: Local, tumour location: f, frontal; p, parietal; t, temporal; o, occipital; th, thalamus; b, basal; c, cerebellum. DUR, duration of symptoms in months. SURV, post-operative survival time in months; survival time between brackets indicates that the patient was alive at closure of the study. NA, data not available.

Genetic data (number of informative markers between brackets): [a]Normalised amplification levels of 12, 11, 26, 8, 33, 11, 15, 15 in tumours 449, 858, 890, 891, 893, 1194, 1666, 1683 respectively. [b]Deletion of part of chromosome arm 17p, including the TP53 gene region. [c]Deletion of D10S25 in non-critical region (Figure 1). [d]Deletion of part of chromosome 10 (Figure 1). [e]Deletion of part of chromosome arm 17p, not including the TP53 gene region (Figure 2).

II. The Student–Newman–Keuls multiple range test revealed a difference between the group of patients showing only LOH 17p and the other three groups. The mean age of these three groups did not differ significantly.

*Genetic alterations in relation to post-operative survival*

Kaplan–Meier survival curves were constructed for the four groups of patients (Figure 3). The median survivals are given in Table II. The survival curves were compared by log-rank test. The resulting $P$-values were adjusted for multiple comparisons by the procedure of Hommel (see Wright, 1992). The difference in median survival between the group of patients with only LOH 17p and the group of patients with LOH 10 or with LOH 10 + LOH 17p was marginally significant ($P$-values of 0.055 and 0.072 respectively). The difference in survival between the LOH 17p only patients and the patients with LOH 10 + EGFR gene amplification was significant ($P = 0.039$). The groups of patients with LOH 10 only, LOH 10 + LOH 17p and LOH 10 + EGFR gene amplification did not differ significantly in median survival between each other ($P$-value for each comparison $> 0.17$).

*Genetic alterations in relation to other clinical data*

All tumours ($n = 7$) with LOH 17p and no other genetic alterations occupied a frontal (frontal or frontoparietal) position (Table I). This suggests a significant ($P < 0.0001$, $\chi^2$ test) predominance of frontally located tumours in the group of patients with LOH 17p as the sole genetic alteration in the tumour.

**Figure 1** Loss of heterozygosity (LOH) analysis of high-grade astrocytomas with deletions of part of chromosome 10. ●, LOH; O, no LOH; ●, not informative.

**Figure 2** RFLP analysis at loci TP53 and D17S66 in tumour 314. Tumour (T) and corresponding blood leucocyte (L) DNAs were digested with *Bgl*II. The resulting fragments were separated in an 0.8% agarose gel, transferred to a nylon filter and hybridised with pProSP53 (TP53, **a**). After removal of the signal, the filter was rehybridised with LEW502 (D17S66, **b**). Sizes of restriction fragments are given in kbp.

### *Multivariate analysis*

The variables age, grade, duration of symptoms, tumour location, extent of tumour resection and genetic subdivision were analysed for prognostic significance by univariate analysis. The variables grade, duration of symptoms, tumour location and extent of tumour resection did not reach significant levels in this analysis. To explore the effect of age and genetic subdivision on survival, Cox's regression model was used. Elimination of the genetic subdivision was a significant step ($P = 0.026$); elimination of age was not significant ($P = 0.17$).

### Discussion

By using LOH 10, LOH 17p and EGFR gene amplification as genetic parameters, we have subdivided our series of high-grade astrocytomas into four types. Type 1 has LOH 17p without concurrent genetic changes, type 2 has LOH 10 as the sole genetic alteration, type 3 is characterised by both LOH 10 and LOH 17p and type 4 is characterised by LOH 10 and EGFR gene amplification. The mean age at operation was significantly lower for the patients with the type 1 tumours than for the patients with types 2, 3 and 4 tumours (Table II). Using the same genetic parameters, Von Deimling *et al.* (1993) proposed a different subdivision for glioblastomas multiforme into two types. Their type 1 tumours had LOH 17p and no EGFR gene amplification and occurred primarily in younger patients. Their type 2 tumours had EGFR gene amplification and no LOH 17p and occurred primarily in older patients. In the system of Von Deimling *et al.*, the type 1 tumours included those with only LOH 17p and those with both LOH 17p and LOH 10. The mean age of their type 1 patients was 40.5 years. In our study, both genetic variants could not be grouped together. We detected a significant age difference between our type 1 patients (only LOH 17p) and our type 3 patients (LOH 10 + LOH 17p), i.e. 39 *vs* 65 years. A possible explanation for this discrepancy is the relatively small number of tumours that was analysed in both studies. Another explanation may be that both series of

**Figure 3** Kaplan-Meier survival curves for patients whose tumours contained only LOH 17p (■, type 1), only LOH 10 (– –, type 2), LOH 10 + LOH 17p (——, type 3) or LOH 10 + EGFR gene amplification (· · ·, type 4).

**Table II** Mean age at operation and median survival period for groups of patients defined by genetic alterations in the tumour

| *Genetic group* | n | *Mean age (years)* | *Median survival (months)* |
|---|---|---|---|
| LOH 17p | 7 | 39 (25–51) | 17 (6–31 or greater) |
| LOH 10 | 14 | 59 (42–74) | 6 (1–30) |
| LOH 10 + LOH 17p | 6 | 65 (59–71) | 3 (0.5–11) |
| LOH 10 + EGFR amplification | 7 | 54 (44–69) | 2 (0.2–11) |

tumours do not match completely because of the different grading systems that were used (glioblastomas multiforme and grade III + IV astrocytomas).

Our type 1 patients differed from the type 2, 3 and 4 patients with regard to their median survival time, although the difference was only significant ($P = 0.039$) between the type 1 and type 4 patients (Table II and Figure 3). Type 1 patients differ from the type 2, 3 and 4 patients in mean age at operation, and age is an important variable with regard to survival (Burger & Green, 1987; Salford *et al.*, 1988). Therefore, the longer survival in the type 1 patients could be explained by their younger age. However, multivariate analysis revealed that, after allowing for age, the genetic subdivision remained a variable with prognostic significance. In this analysis, age was assumed to be linearly related to the logarithm of the hazard rate. If this assumption is not correct, then, owing to the lack of overlap in age between the type 1 patients and the other groups of patients, no adjustment for age would be possible.

Comparable studies with regard to survival in patients with high-grade astrocytomas and with the same set of genetic alterations have, to our knowledge, not been published. Bigner *et al.* (1988) studied EGFR gene amplification in malignant gliomas but could not detect a significant effect on median survival. In contrast to this, Hurtt *et al.* (1992) reported a significant and age-independent decrease in median survival in patients with EGFR gene amplification. If we consider EGFR gene amplification as an independent variant in Cox's regression model, then it has age-corrected prognostic significance ($P = 0.004$). However, as noted by Von Deimling *et al.* (1992*b*) and as shown in Table I, EGFR gene amplification is strongly associated with LOH 10. Our type 4 patients with LOH 10 + EGFR gene amplification had a similar mean age to the type 2 patients with LOH 10 only (59 and 54 years respectively). The difference in survival between both groups of patients is not significant ($P = 0.17$), thereby questioning the prognostic importance of EGFR gene amplification. Thus, the contradictory reports on the prognostic importance of EGFR gene amplification could be caused by differences in the chromosome 10 status of the tumours that were examined. In all our type 1 patients, the tumour occupied a frontal position. As noted before, these patients were relatively young at presentation and had a favourable prognosis. This observation genetically supports other clinical studies reporting a longer survival time for patients with frontally located lesions (North *et al.*, 1990; Curran *et al.*, 1992; Ayoubi *et al.*, 1993).

In summary, our data suggest that histopathologically high-grade astrocytomas constitute a genetically heterogeneous group of tumours. They can be subdivided by using allele loss on chromosomes 10 and 17p and EGFR gene amplification as genetic parameters. The genetic subdivision into four types correlates with mean age and median survival of the different groups of patients. Under certain assumptions, the genetic subdivision has major prognostic significance. The discrepancies between the results presented here and those of others may in part be explained by the small sample sizes in the studies.

We are grateful to Professor C.A.F. Tulleken and Dr L. van de Ven (Neurosurgery Department, State University, Utrecht) and the surgeons of the Neurosurgery Department of the Academic Medical Centre in Amsterdam for kindly supplying the tumour and blood samples. We thank N. Claessen (Department of Neuropathology, University of Amsterdam) for histopathological procedures and J. Juijn for excellent technical assistance.

### References

AYOUBI, S., WALTER, P.H., NAIK, S., SANKARAN, M. & ROBINSON, D. (1993). Audit in the management of gliomas. *Br. J. Neurosurg.*, **7**, 61–69.

BAKER, S.J., PREISINGER, A.C., JESSUP, J.M., PARASKEVA, C., MARKOWITZ, S. & WILLSON, J.K. (1990). p53 gene mutations occur in combination with 17p allelic deletions as late events in colorectal tumorigenesis. *Cancer Res.*, **50**, 7717–7722.

BIGNER, S.H., BURGER, P.C., WONG, A.J., WERNER, M.H., HAMILTON, S.R., MUHLBAIER, L.H., VOGELSTEIN, B. & BIGNER, D.D. (1988). Gene amplification in malignant human gliomas: clinical and histopathologic aspects. *J. Neuropathol. Exp. Neurol.*, **47**, 191–205.

BURGER, P.C. & GREEN, S.B. (1987). Patient age, histologic features, and length of survival in patients with glioblastoma multiforme. *Cancer*, **59**, 1617–1625.

BIJLSMA, E.K., LEENSTRA, S., WESTERVELD, A., BOSCH, D.A. & HULSEBOS, T.J.M. (1994). Amplification of the anonymous marker D17S67 in malignant astrocytomas. *Genes Chrom. Cancer*, **9**, 148–152.

CHUNG, R., WHALEY, J., KLEY, N., ANDERSON, K., LOUIS, D., MENON, A., HETTLICH, C., FREIMAN, R., HEDLEY-WHYTE, E.T., MARTUZA, R., JENKINS, R., YANEDELL, D. & SEIZINGER, B.R. (1991). TP53 gene mutations and 17p deletions in human astrocytomas. *Genes Chrom. Cancer*, **3**, 323–331.

COLLINS, V.P. & JAMES, C.D. (1993). Gene and chromosomal alterations associated with the development of human gliomas. *FASEB J.*, **7**, 926–930.

CURRAN Jr, W.J. SCOTT, C.B., HORTON, J., NELSON, J.S., WEINSTEIN, A.S., NELSON, D.F., FISCHBACH, A.J., CHANG, C.H., ROTMAN, M., ASBELL, S.O. & POWLIS, W.D. (1992). Does extent of surgery influence outcome for astrocytoma with atypical or anaplastic foci (AAF)? A report from three Radiation Therapy Oncology Group (RTOG) trials. *J. Neurooncol.*, **12**, 219–227.

DAUMAS-DUPORT, C., SCHEITHAUER, B., O'FALLON, J. & KELLY, P. (1988). Grading of astrocytomas. *Cancer*, **62**, 2152–2165.

EKSTRAND, A.J., JAMES, C.D., CAVENEE, W.K., SELIGER, B., PETTERSSON, R.F. & COLLINS, V.P. (1991). Genes for epidermal growth factor receptor, transforming growth factor alpha, and epidermal growth factor and their expression in human gliomas in vivo. *Cancer Res.*, **51**, 2164–2172.

EL-AZOUZI, M., CHUNG, R.Y., FARMER, G.E., MARTUZA, R.L., BLACK, P.M., ROULEAU, G.A., HETTLICH, C., HEDLEY-WHYTE, E.T., ZERVAS, N.T., PANAGOPOULOS, K., NAKAMURA, Y., GUSELLA, J.F. & SEIZINGER, B.R. (1989). Loss of distinct regions on the short arm of chromosome 17 associated with tumorigenesis of human astrocytomas. *Proc. Natl Acad. Sci. USA*, **86**, 7186–7190.

FEINBERG, A.P. & VOGELSTEIN, B.A. (1984). Addendum. A technique for radiolabelling DNA restriction endonuclease fragments to high specific activity. *Anal. Biochem.*, **137**, 266–267.

FRANKEL, R.H., BAYONA, W., KOSLOW, M. & NEWCOMB, E.W. (1992). p53 mutations in human malignant gliomas: comparison of loss of heterozygosity with mutation frequency. *Cancer Res.*, **52**, 1427–1433.

FUJIMOTO, M., FULTS, D.W., THOMAS, G.A., NAKAMURA, Y., HEILBRUN, M.P., WHITE, R., STORY, J.L., NAYLOR, S.L., KAGAN-HALLET, K.S. & SHERIDAN, P.J. (1989). Loss of heterozygosity on chromosome 10 in human glioblastomas multiforme. *Genomics*, **4**, 210–214.

FULTS, D., TIPPETS, R.H., THOMAS, G.A., NAKAMURA, Y. & WHITE, R. (1989). Loss of heterozygosity for loci on chromosome 17p in human malignant astrocytoma. *Cancer Res.*, **49**, 6572–6577.

FULTS, D., PEDONE, C.A., THOMAS, G.A. & WHITE, R. (1990). Allelotype of human malignant astrocytoma. *Cancer Res.*, **50**, 5784–5789.

FULTS, D., BROCKMEYER, D., TULLOUS, M.W., PEDONE, C.A. & CAWTHON, R.M. (1992). p53 mutation and loss of heterozygosity on chromosomes 17 and 10 during human astrocytoma progression. *Cancer Res.*, **52**, 674–679.

FULTS, F. & PEDONE, C. (1993). Deletion mapping of the long arm of chromosome 10 in glioblastoma multiforme. *Gene Chrom. Cancer*, **7**, 173–177.

HUMAN GENE MAPPING 11 (1991). *Cytogenet. Cell Genet.*, **58**, 1–2200.

HOLLSTEIN, M., SIDRANSKY, D., VOGELSTEIN, B. & HARRIS, C.C. (1991) p53 mutations in human cancers. *Science*, **253**, 49–53.

HURTT, M.R., MOOSSY, J., DONOVAN-PELUSO, M. & LOCKER, J. (1992). Amplification of epidermal growth factor receptor gene in gliomas: histopathology and prognosis. *J. Neuropathol. Exp. Neurol.*, **51**, 84-90.

JAMES, C.D., CARLBOM, E., DUMANSKI, J.P., HANSEN, M., NORDENSKJÖLD, M., COLLINS, V.P. & CAVENEE, W.K. (1988). Clonal genomic alterations in glioma malignancy stages. *Cancer Res.*, **48**, 5546-5551.

JAMES, C.D, CARLBOM, E., NORDENSKJÖLD, M., COLLINS, V.P. & CAVENEE, W.K. (1989). Mitotic recombination of chromosome 17 in astrocytomas. *Proc. Natl Acad Sci USA*, **86**, 2858-2862.

KARLBOM, E.A., JAMES, C.D., BOETHIUS, J., CAVENEE, W.K., COLLINS, V.P., NORDENSKJÖLD, M. & LARSSON, C. (1993). Loss of heterozygosity in malignant gliomas involves at least three distinct regions on chromosome 10. *Hum. Genet.*, **92**, 169-174.

KERNOHAN, J.W. & SAYRE, G.P. (1952). *Tumors of the Central Nervous System.* Armed Forces Institute of Washington: Washington, DC.

LEENSTRA, S., TROOST, D., WESTERVELD, A., BOSCH, D.A. & HULSEBOS, T.J.M. (1992). Molecular characterization of areas with low grade tumor or satellitosis in human malignant astrocytomas. *Cancer Res.*, **52**, 1568-1572.

MATLASHEWSKI, G.J., TUCK, S., PIM, D., LAMB, P., SCHNEIDER, J. & CRAWFORD, L.V. (1987). Primary structure polymorphism at amino acid residue 72 of human p53. *Mol. Cell. Biol.*, **7**, 961-963.

MATSUNAMI, N., SMITH, B., BALLARD, L., LENSCH, M.W., ROBERTSON, M., ALBERTSEN, H., HANEMANN, C.O., MÜLLER, H.W., BIRD, T.D., WHITE, R. & CHANCE, P.F. (1992). Peripheral myelin protein-22 gene maps in the duplication in chromosome 17p11.2 associated with Charcot-Marie-Tooth 1A. *Nature Genet.*, **1**, 176-179.

NIGRO, J.M., BAKER, S.J., PREISINGER, A.C., JESSUP, J.M, HOSTETTER, R., CLEARY, K. & BIGNER, S.H. (1989). Mutations in the p53 gene occur in diverse human tumour types. *Nature*, **342**, 705-708.

NORTH, B., REILLY, P., BLUMBERGS, P., RODER, D. & ESTERMAN, A. (1990). Malignant astrocytoma in South Australia: treatment and case survival. *Med. J. Aust.*, **153**, 250-254.

PENTAO, L., WISE, C.A., CHINAULT, A.C., PATEL, P.I. & LUPSKI, J.R. (1992). Charcot-Marie-Tooth type 1A duplication appears to arise from recombination at repeat sequences flanking the 1.5 Mb monomer unit. *Nature Genet.*, **2**, 292-300.

RANSOM, D.T., RITLAND, S.R., MOERTEL, C.A., DAHL, R.J., O'FALLON, J.R., SCHEITHAUER, B.W., KIMMEL, D.W., KELLY, P.J., OLOPADE, O.I., DIAZ, M.O. & JENKINS, R.B. (1992). Correlation of cytogenetic analysis and loss of heterozygosity studies in human diffuse astrocytomas and mixed oligo-astrocytomas. *Genes Chrom. Cancer*, **5**, 357-374.

RASHEED, B.K.A., FULLER, G.N., FRIEDMAN, A.H., BIGNER, D.D. & BIGNER, S.H. (1992). Loss of heterozygosity for 10q loci in human gliomas. *Genes Chrom. Cancer*, **5**, 75-82.

REVESZ, T., SCARAVILLI, F., COUTINHO, L., COCKBURN, H., SACARES, P. & THOMAS, D.G.T. (1993). Reliability of histological diagnosis including grading in gliomas biopsied by image-guided stereotactic technique. *Brain*, **116**, 781-793.

SALFORD, L.F., BRUN, A. & NIRFALK, S. (1988). Ten-year survival among patients with supratentorial astrocytomas grade III and IV. *J. Neurosurg.*, **69**, 506-509.

SCANLON, P.W. & TAYLOR, W.F. (1979). Radiotherapy of intracranial astrocytomas: analysis of 417 cases treated from 1960 through 1969. *Neurosurgery*, **5**, 301-308.

TROELSTRA, C., LANDSVATER, R.M., WIEGANT, J., VAN DER PLOEG, M., VIEL, G., BUYS, C.H.C.M. & HOEIJMAKERS, J.H.J. (1992). Localization of the nucleotide excision repair gene ERCC6 to human chromosome 10q11-q21. *Genomics*, **12**, 745-749.

VENTER, D.J. & THOMAS, D.G. (1991). Multiple sequential molecular abnormalities in the evolution of human gliomas. *Br. J. Cancer*, **63**, 753-757.

VON DEIMLING, A., EIBL, R.H., OHGAKI, H., LOUIS, D.N., VON AMMON, K., PETERSEN, I., KLEIHUES, P., CHUNG, R.Y., WIESTLER, O.D. & SEIZINGER, B.R. (1992*a*). p53 mutations are associated with 17p allelic loss in grade II and grade III astrocytoma. *Cancer Res.*, **52**, 2987-2990.

VON DEIMLING, A., LOUIS, D.N., VON AMMON, K., PETERSEN, I., HOELL, T., CHUNG, R.Y., MARTUZA, R.L., SCHOENFELD, D.A., YASARGIL, M.G. & WIESTLER, O.D. (1992*b*). Association of epidermal growth factor receptor gene amplification with loss of chromosome 10 in human glioblastoma multiforme. *J. Neurosurg.*, **77**, 295-301.

VON DEIMLING, A., VON AMMON, K., SCHOENFELD, D., WIESTLER, O.D., SEIZINGER, B.R. & LOUIS, D.N. (1993). Subsets of glioblastoma multiforme defined by molecular genetic analysis. *Brain Pathol.*, **3**, 19-26.

WATANABE, K., NAGAI, M., WAKAI, S., ARAI, T. & KAWASHIMA, K. (1990). Loss of constitutional heterozygosity in chromosome 10 in human glioblastoma. *Acta Neuropathol.*, **80**, 251-254.

WONG, A.J., BIGNER, S.H., BIGNER, D.D., KINZLER, K.W., HAMILTON, S.R. & VOGELSTEIN, B. (1987). Increased expression of the epidermal growth factor receptor gene in malignant gliomas is invariably associated with gene amplification. *Proc. Natl Acad. Sci. USA*, **84**, 6899-6903.

WRIGHT, S.P. (1992). Adjusted p-values for simultaneous inference. *Biometrics*, **48**, 1005-1013.